# Clinical efficacy and satisfaction of a digital wheeze detector in a multicentre randomised controlled trial: the WheezeScan study

**DOI:** 10.1183/23120541.00518-2023

**Published:** 2024-01-15

**Authors:** Yen Hoang Do, Wim van Aalderen, Ellen Dellbrügger, Claude Grenzbach, Jonathan Grigg, Ulrike Grittner, Eric Haarman, Camilo José Hernandez Toro, Bulent Karadag, Siri Roßberg, Tina-Maria Weichert, Abigail Whitehouse, Antonio Pizzulli, Paolo Maria Matricardi, Stephanie Dramburg

**Affiliations:** 1 Department of Pediatric Respiratory Medicine, Immunology and Critical Care Medicine, Charité – Universitätsmedizin Berlin, Berlin, Germany; 2 Department of Pediatric Respiratory Medicine and Allergy, Emma Children's Hospital, Amsterdam UMC, University of Amsterdam, Amsterdam, The Netherlands; 3 Pediatric Pulmonologist, Berlin, Germany; 4 Centre for Genomics and Child Health, Blizard Institute, Queen Mary University of London, London, UK; 5 Institute of Biometry and Clinical Epidemiology, Charité – Universitätsmedizin Berlin, Berlin, Germany; 6 Division of Pediatric Pulmonology, Marmara University, Istanbul, Turkey

## Abstract

**Introduction:**

Wheezing is common in preschool children and its clinical assessment often challenging for caretakers. This study aims to evaluate the impact of a novel digital wheeze detector (WheezeScan™) on disease control in a home care setting.

**Methods:**

A multicentre randomised open-label controlled trial was conducted in Berlin, Istanbul and London. Participants aged 4–84 months with a doctor's diagnosis of recurrent wheezing in the past 12 months were included. While the control group followed usual care, the intervention group received the WheezeScan™ for at-home use for 120 days. Parents completed questionnaires regarding their child's respiratory symptoms, disease-related and parental quality of life, and caretaker self-efficacy at baseline (T0), 90 days (T1) and 4 months (T2).

**Results:**

A total of 167 children, with a mean±sd age of 3.2±1.6 years, were enrolled in the study (intervention group n=87; control group n=80). There was no statistically significant difference in wheeze control assessed by TRACK (mean difference 3.8, 95% CI −2.3–9.9; p=0.2) at T1 between treatment groups (primary outcome). Children's and parental quality of life and parental self-efficacy were comparable between both groups at T1. The evaluation of device usability and perception showed that parents found it useful.

**Conclusion:**

In the current study population, the wheeze detector did not show significant impact on the home management of preschool wheezing. Hence, further research is needed to better understand how the perception and usage behaviour may influence the clinical impact of a digital support.

## Tweetable abstract

**In this study population, a digital wheeze detector did not show significant impact on the management of preschool wheezing disorders at home. Further research is needed to enhance device usability and understand parental perception of this device.** https://bit.ly/45Wgazm

## Introduction

Wheezing caused by viral infections is common among preschool children, with up to 50% experiencing at least one wheezing episode in the first 6 years of their life [1, 2]. Studies have shown that children suffering from wheeze, especially persistent phenotypes, have a higher risk of developing asthma later in childhood [3]. The main treatment goals for preschool wheezing are respiratory symptom control, reduction of exacerbations and increasing quality of life for the child [4]. To achieve these aims, training caregivers to correctly detect airway obstruction and administer pharmacological and supportive treatments is essential. However, when it comes to young children (under the age of 6 years), the correct assessment of wheezing breath sounds is particularly difficult, since the distinction of wheezing sounds from physiological breath sounds may be challenging [5, 6]. A misjudgement of respiratory symptoms may consequently result in an under- or overtreatment with reliever medication [7, 8]. On the other hand, it has been shown that guided self-management options for respiratory diseases such as wheezing can reduce hospitalisations and visits to the emergency department and improve lung function [9, 10]. Therefore, supporting parents in the at-home self-management of their child's respiratory condition is key to obtaining and maintaining respiratory symptom control and improve patient outcomes.

Over the past years, adoption of digital health technologies has continuously increased, from patient-specific electronic health records over sensor technology and (adherence) monitors to telemedicine options for easier accessibility to healthcare specialists [11, 12]. Digital solutions are often easily available and adaptive into everyday life, due to the increasing access to portable internet and smartphones around the globe [13]. Mobile health also allows an efficient and cost-effective data collection *via* electronic diaries and is usually well received by both patients and clinicians [11]. Therefore, there is a growing interest in the potential of eHealth solutions to allow tailored self-management options specifically for young children suffering from wheezing disorders as well as their parents.

There are several devices such as portable smart inhalers [14, 15], asthma symptom diaries [16, 17], wearable trackers [18] and health-education-based computer games [19] available for the at-home management of wheezing disorders. Despite progress in the development and testing of medication sensors and adherence reminders, only a few devices have been built and evaluated to detect pathological airway sounds such as wheezing [20, 21]. While most digital support tools are currently being evaluated in feasibility studies and small clinical trials, large randomised controlled trials testing their clinical impact on outcomes such as symptom control and quality of life are scarce. In terms of sound recognition, it is not only important to test the accuracy of devices, but also to evaluate the usability and clinical efficacy. These aspects are particularly important to determine potential benefits and limitations, ensuring the safe and effective use of digital support for conditions such as preschool wheezing and paediatric asthma.

The aim of this study was to test the hypothesis that a digital support tool for wheeze recognition improves symptom control in a study population of preschool children. In addition, the device's impact on disease-specific and parental quality of life as well as subjective self-efficacy in disease management among parents was evaluated in this randomised controlled trial across culturally diverse paediatric populations. Finally, the usability of and satisfaction with the device were assessed.

## Materials and methods

### Study population

The multicentre randomised controlled open-label trial consisted of two groups, following usual care with (intervention) and without (control) the use of a digital wheeze detector (WheezeScan™; OMRON Healthcare Co. Ltd, Kyoto, Japan) device, respectively. Patients were recruited between October 2021 and September 2022 in six specialised paediatric pulmonology outpatient departments located in Berlin (ambulatory clinics of E. Dellbrügger, S. Roßberg, T. Weichert and C. Grenzbach), London (Blizard Institut at Queen Mary University of London) and Istanbul (ambulatory clinic of Karadag and Marmara University Istanbul). The inclusion criteria were: 1) age 4 months to 7 years; 2) at least one episode of doctor-diagnosed wheezing and/or recurrent cough requiring treatment according to Global Initiative for Asthma (GINA) guidelines steps 1 or 2 in the last 12 months [22]; and 3) availability of a smartphone. The exclusion criteria were: 1) an anatomical malformation causing chronic nasal and/or bronchial obstruction; 2) presence of another severe chronic disease; and 3) wheezing disorders requiring treatment step 3 or 4 according to GINA guidelines. The study was registered in the German Clinical Trials Registry (DRKS00026740), and ethics approval was obtained at all study sites.

### Study design

**FIGURE 1:**
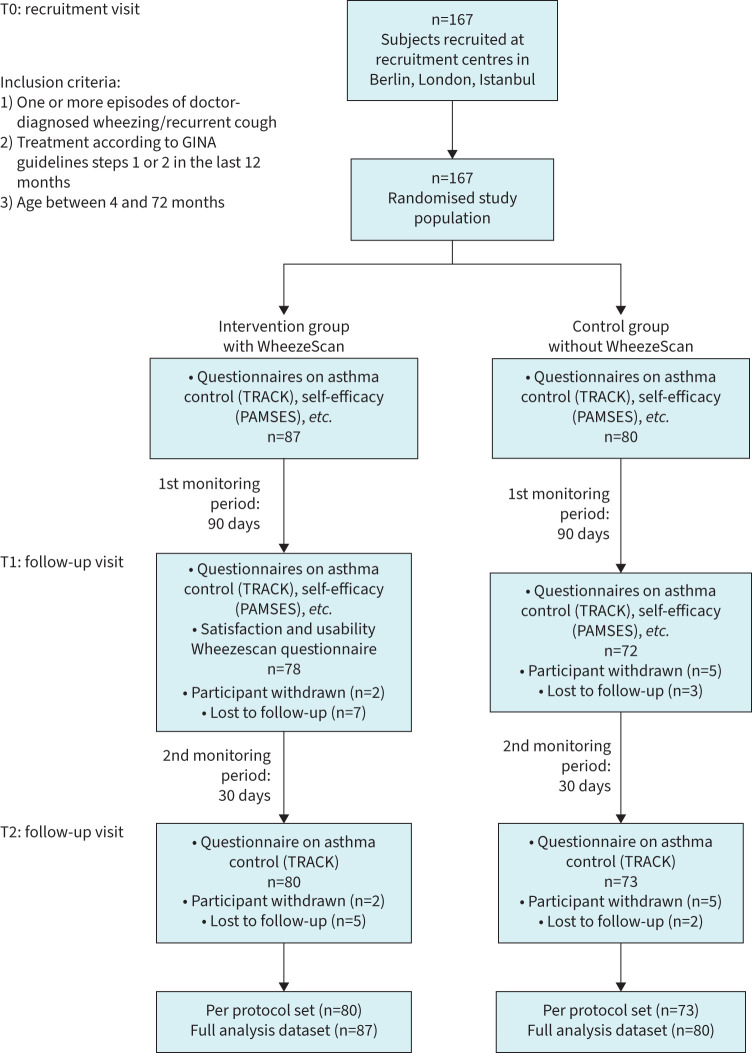
Consort chart with inclusion criteria, recruitment, study design and timeline. GINA: Global Initiative for Asthma.

After randomisation, parents were interviewed regarding the personal and family anamnesis of their child including information on allergic diseases and demographic variables. Validated questionnaires regarding disease severity and disease-specific quality of life were administered. Families of the intervention group were trained to use the digital wheeze detector WheezeScan™ and received their device to take home. A comparison between the device's measurement and the study physician's auscultation was recorded. The current treatment scheme was reported for all participants by the study physician. After the recruitment visit (T0), all participating families were asked to fill in an electronic daily questionnaire *via* the mobile application WheezeMonitor® (TPS Production S.rl, Rome, Italy) over a total of 120 observation days. Families of the intervention group were additionally encouraged to use the WheezeScan™ device whenever they felt that their child could be experiencing respiratory distress. An initial follow-up visit (T1) was performed after 90 days of the monitoring period. Parents responded again to questionnaires on disease severity, self-efficacy and quality of life, and the attending physician assessed whether an adaptation of treatment was necessary according to GINA guidelines. Participants of the intervention group were also asked to fill a usability and satisfaction questionnaire regarding the WheezeScan™ device. After T1, all participants continued the observation period for another 30 days until the final study visit (T2), where any changes in asthma control *via* the TRACK (Test for Respiratory and Asthma Control in Kids) questionnaire and changes in treatment according to GINA guidelines were recorded. The aim of the final visit was to evaluate potential clinical improvements after a treatment adaptation. For an overview on the enrolment and randomisation, please see figure 1.

### Questionnaires

The primary outcome of symptom control was assessed *via* the TRACK [23] questionnaire at T1. Secondary outcomes were assessed at T1 *via* the Parental Asthma Management Self-Efficacy Scale (PAMSES) [24], the Pediatric Asthma Caregiver's Quality of Life Questionnaire (PACQLQ) [25] and the TAPQOL on parent's perception of health-related quality of life in preschool children [26], and user satisfaction with the device in the intervention group was assessed *via* a usability and satisfaction questionnaire. An additional secondary outcome was assessed *via* the TRACK questionnaire at T2. The secondary outcome regarding the frequency of reliever medication use was recorded in the e-diary of the monitoring App during the entire study period.

### Digital wheeze detector

Families allocated to the intervention group received a digital wheeze detector (WheezeScan™, OMRON Healthcare Co. Ltd, Kyoto, Japan) to be used whenever needed in a home care setting. Performance, safety and usability of the device had been evaluated in previous studies [27, 28]. The WheezeScan™ assesses respiratory symptoms of the child *via* a sound detector once placed on the chest just below the right collarbone. After approximately 30 s of measurement, results are indicated *via* an integrated display (supplementary figure S1).

### Statistics

For information on the sample size calculation, please see the supplementary material. As descriptive analysis, summary measures such as mean±sd, median, first and third quartile (q1–q3), number (n) and percentage (%) depending on the scaling of the variables are reported for the two groups and the total study population. The intervention effect on the primary and secondary outcomes was assessed in the full analysis set using the intention-to-treat principle. All randomised participants were included in the full analysis set.

Primary analysis consisted of a comparison of the TRACK score at T1 between intervention and control group using ANCOVA with the TRACK score at T1 as dependent variable and treatment group, centre and TRACK score at T0 as covariates. Mean difference between intervention and control group and 95% CI as well as standardised effect size were estimated. A two-sided significance level of α=0.05 was used for the primary analysis. For information on the secondary and subgroup analyses, please see the supplementary material. All statistical analyses were performed in R version 4.2.2 (R Foundation for Statistical Computing).

More information on materials and methods can be found in the supplementary material.

## Results

In total, 167 children were enrolled in the study, with 85 (50.9%) of the children being recruited in Berlin, 59 (35.3%) in Istanbul, and 23 (13.8%) in London. 87 participants were allocated to the intervention group, and 80 to the control group. Of the 167 families, 150 (89.8%) completed the first follow-up assessment and 153 (91.6%) the final study visit. While seven participants were lost to follow-up, another seven actively withdrew their consent owing to experiencing technical difficulties with the device and/or monitoring application. On average, participants had a mean age of 3.2±1.6 years, 116 of 167 (69.5%) were male and 133 of 167 (79.6%) had one or more siblings. Relevant differences between the two treatment groups were observed regarding the presence of an allergic disease. While 31% (27 of 87) of the children in the intervention group suffered from allergy, this was only the case for 17.5% (14 of 80) of the controls (table 1).

**TABLE 1:** Baseline characteristics

	**Overall**	**Intervention**	**Control**
**Patients n**	167	87	80
**Sex (male)**	116(69.5)	65(74.7)	51(63.7)
**Age years**	3.2±1.6	3.1±1.6	3.5±1.6
**Patient distribution by centre**			
Berlin	85(50.9)	44(50.6)	41(51.2)
Istanbul	59(35.3)	31(35.6)	28(35.0)
London	23(13.8)	12(13.8)	11(13.8)
**Number of siblings**			
0	34(20.4)	15(17.2)	19(23.8)
1	78(46.7)	47(54.0)	31(38.8)
2	40(24.0)	17(19.5)	23(28.7)
3	12(7.2)	7(8.0)	5(6.2)
4	1(0.6)	1(1.1)	0(0.0)
5	2(1.2)	0(0.0)	2(2.5)
**Week of pregnancy at birth**	38.6±2.5	38.5±2.9	38.7±2.1
**Body weight at birth g**	3350±640	3400±677	3300±596
**Body height at birth cm**	50.8±3.8	50.8±4.2	50.9±3.3
**Allergic comorbidities**			
Any allergy	41(24.6)	27(31)	14(17.5)
Allergic rhinoconjunctivitis	9(5.4)	5(5.7)	4(5.0)
Atopic dermatitis	8(4.8)	6(6.9)	2(2.5)
Food allergy	8(4.8)	4(4.6)	4(5.0)
Other allergy	8(4.8)	7(8.0)	1(1.3)
**Allergy diagnostics**			
Positive SPT and/or serum IgE test	47(28.1)	32(36.8)	15(18.8)
**Family history of allergies**			
Father allergic	53(31.7)	34(39.1)	19(23.8)
Mother allergic	38(22.8)	25(28.7)	13(16.2)
Allergic sibling	23(13.8)	16(18.4)	7(8.8)

Data are presented as n (%) or mean±sd. SPT: skin prick test.

### Impact of the digital wheeze detector on asthma control (TRACK): primary outcome analysis

**FIGURE 2:**
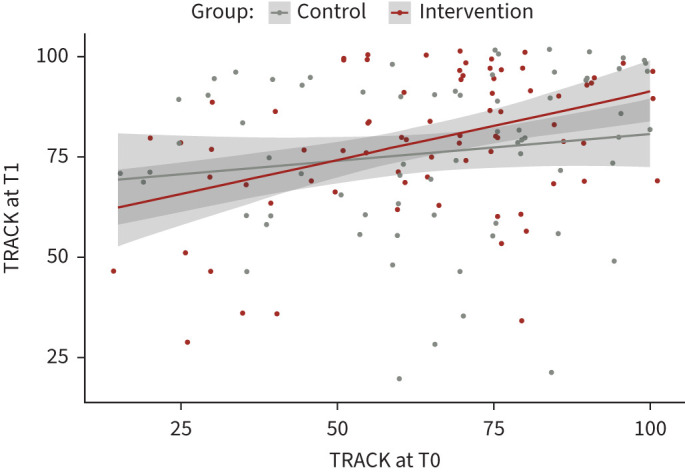
Individual measures of wheeze control (TRACK) at baseline (T0: baseline) and follow-up (T1: 90 days) for the intervention (n=78) and control group (n=72).

The intervention group started off with a slightly lower baseline TRACK score (64.5±20.9 points) than the control group (66.3±22.8 points). At T1, the intervention group had an average of 79.1±17.7 points, while the control group had a mean score of 76.2±19.8 points (table 2). Although the absolute increase in the intervention group was higher than in the control group (figure 2), the mean difference (at T1) between the two groups was not statistically significant (3.6, 95% CI −2.3–9.4, p=0.228, primary end-point). At T2 (120 days after baseline), both groups had a similar mean TRACK score (the intervention group: 78.4±18.7 points, the control group: 78.3±19.4 points) (supplementary figure S3A). Regarding a potential impact of treatment changes at T1, no changes in treatment were performed.

**TABLE 2:** Primary and secondary outcomes by study group and time point (T1=90 days)

	**All participants**		**Intervention group**		**Control group**		**Difference** **intervention *versus* control group (95% CI)**
	**T0**	**T1**	**T0**	**T1**	**T0**	**T1**	
**Participants n**	167	150	87	78	80	72	
**TRACK** **^#^**	65.4±21.8	77.7±18.7	64.5±20.9	79.1±17.7	66.3±22.8	76.2±19.8	3.59 (−2.26–9.44); p=0.23
**PAMSE**	49.9±8.8	51.0±9.4	50.5±8.6	51.1±8.9	49.1±9.0	51.0±10.0	−0.80 (3.64–2.04); p=0.58
**PACQLQ**	5.5±1.3	6.3±1.0	5.6±1.3	6.4±1.0	5.5±1.4	6.1±1.0	0.24 (−0.07–0.55); p=0.12
**TAPQOL**	62.9±11.0	68.1±11.2	62.6±12.0	67.2±12.8	63.1±9.9	69.1±9.1	−0.91 (−4.12–2.31); p=0.58

Data are presented as mean±sd unless indicated otherwise. Calculation of all intervention effects were based on multiple imputation estimates and ANCOVA. ^#^: primary end-point.

**FIGURE 3:**
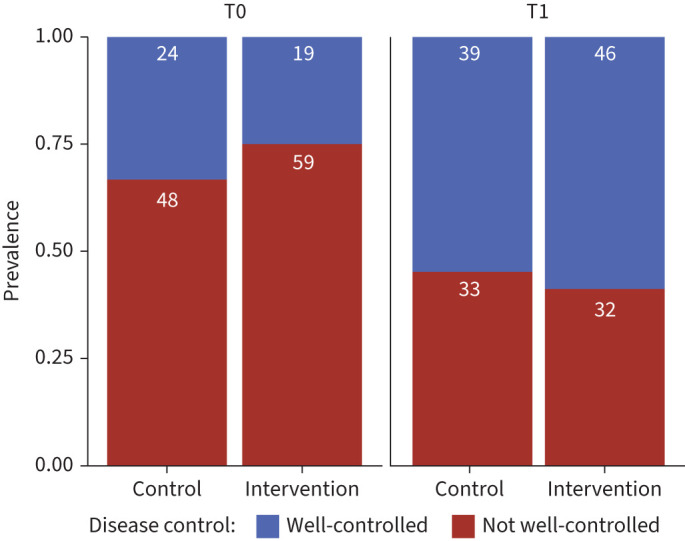
Proportion of well-controlled (TRACK ≥80 points) and not well-controlled (TRACK <80 points) by study group (control group n=80, intervention group n=87) and time point. Total number of participants in each TRACK group per treatment group per time point are shown at the top of each section of the bar plot.

When analysing categorical asthma control (“not well controlled” and “well controlled”) between T0 and T1 in each of the treatment groups, the proportion of patients with good control of wheezing increased in both groups with a slightly larger increase among the intervention group (increase in portion of participants with controlled wheezing from T0 to T1 in control group by 15%, in intervention group by 27%) (figure 3). For subgroup analyses please refer to the supplementary material.

### Disease-specific and parental quality of life and parental self-efficacy in asthma management

**FIGURE 4:**
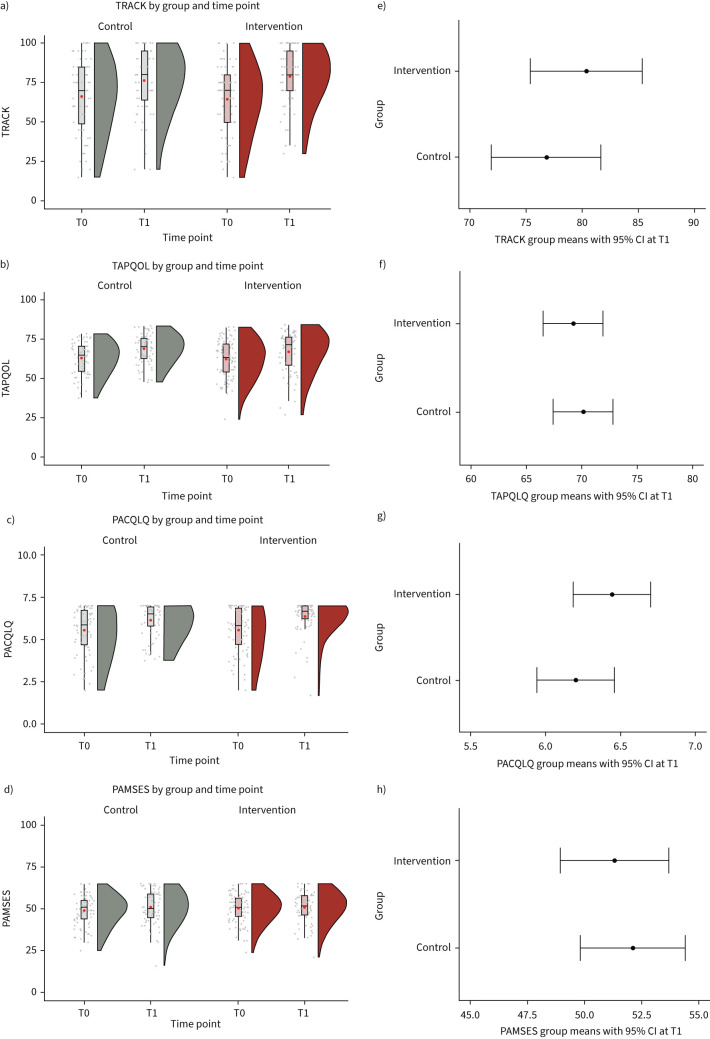
a–d) Wheeze control (TRACK), disease-specific quality of life (TAPQOL), parental quality of life (PACQLQ) and parental asthma management self-efficacy (PAMSES) by study groups (control group n=80, intervention group n=87) and time points (T0: baseline and T1: 90 days) depicted as box plots/rain cloud plots. e–h) Additionally, estimated marginal means using linear regression models are shown by group at T1 (90 days) with 95% CI.

The disease-specific quality of life of the participating children improved in both groups between the baseline and follow-up visit. There was no intervention effect at T1, as underlined by the mean difference between groups at T1 of −0.9 (95% CI −4.1–2.3, p=0.6) (figure 4b and f). Similar results were observed for parental quality of life (PACQLQ mean difference 0.2, 95% CI −0.1–0.6; p=0.1) (figure 4c and g) and parental self-efficacy in managing their child's wheezing condition (PAMSE score mean difference −0.8, 95% CI −3.6–2.0; p=0.6) (figure 4d and h, table 2). Interestingly, all secondary outcomes improved between study visits with little to no differences between the study groups.

### Use of reliever medication and unscheduled healthcare utilisation

In case of short-acting β-agonist treatment, no pronounced differences were observed between study arms (supplementary figure S8). Further, the number of unscheduled visits at the paediatrician's office or emergency department between T0 and T1 (120 days) was slightly higher in the control group than in the intervention group with a mean of 2.16±3.33 *versus* 1.57±2.45 and 0.81±1.34 *versus* 0.60±1.73 visits, respectively (Table 3).

**TABLE 3:** Number of days with wheezing episodes and unscheduled healthcare utilisation between T0 and T1 (120 days) per study group

	**Intervention**	**Control**
**Participants n**	87	80
**Days with little wheezing**	7.9±10.3	7.8±11.9
**Days with strong wheezing**	0.9±2.5	1.4±4.9
**Unscheduled doctor's visit**	1.6±2.5	2.2±3.3
**Visit to an emergency department**	0.6±1.7	0.8±1.3

Data are presented as mean±sd unless indicated otherwise.

### Satisfaction and usability

Regarding device usability, 80% (60 of 75) of the intervention group indicated having used the digital wheeze detector without complications and only 14 of 75 (19%) considered the handling difficult. If parents experienced problems, these were most frequently due to challenges in keeping the child calm enough for the device to function well. Ten families (13%) of the intervention group reported having difficulties in technically operating the device (table 4]) When asked whether they believe their child to have benefitted from the use of the WheezeScan™, the majority answered positively (45 of 75, 60%). For more information on usability results by study centre and age group, please see the supplementary material.

**TABLE 4:** Satisfaction and device usability among different age groups of the intervention group at T1

	**4–12 months**	**13–48 months**	**>48 months**
**Participants n**	17	43	15
**How was the handling of the WheezeScan at home?**			
Without complications	13 (76)	32 (74)	15 (100)
Difficult	3 (18)	11 (26)	0 (0)
Impossible	1 (6)	0 (0)	0 (0)
**If there were problems in use, what were they most likely to be?**			
Difficulties in operating the device	2 (12)	5 (12)	3 (20)
Child did not tolerate the measurements	4 (24)	11 (26)	4 (27)
No problems occurred	9 (53)	24 (56)	8 (53)
No answer possible	2 (12)	3 (7)	0 (0)
**Do you think your child benefitted from using the device?**			
Yes	6 (35)	15 (35)	10 (67)
Rather yes	5 (29)	8 (19)	1 (7)
Rather no	2 (12)	9 (21)	3 (20)
No	4 (24)	11 (26)	1 (7)
**Would you recommend the device to other parents?**			
Yes	10 (67)	17 (40)	9 (53)
Rather yes	2 (13)	8 (19)	2 (12)
Rather no	0 (0)	10 (23)	2 (12)
No	3 (20)	8 (19)	4 (24)
**Are you interested in continuing to use the device?**			
Yes	10 (59)	16 (37)	8 (53)
Rather yes	0 (0)	8 (19)	3 (20)
Rather no	1 (6)	6 (14)	0 (0)
No	6 (35)	13 (30)	4 (27)

Data are presented as n (%).

## Discussion

In our multicentre randomised open-label controlled trial on the clinical efficacy and usability of a digital wheeze detector for preschool children we observed: 1) no significant difference regarding wheeze control between study groups; 2) no impact on disease-specific or parental quality of life; 3) almost no differences regarding parental self-efficacy in managing the child's disease; 4) good usability reports by parents, particularly for the older children in our study; 5) positively perceived benefit for the child from device usage, particularly for the very young and older children; and 6) regional differences in device usage and evaluation.

### Performance of digital interventions for wheezing disorders

Although often limited by small patient numbers and exploratory designs, several studies have reported positive results on the sensitivity and specificity of electronic devices detecting pathological airway sounds such as wheezing or cough [20, 21, 27, 29, 30]. For instance, the device used in this study demonstrated high sensitivity (100%) and specificity (95.7%) for wheeze detection when compared to the auscultation of specialised physicians [27]. A study, comparing digital electronic stethoscopes to paediatricians’ auscultation has even suggested that the digital stethoscopes tested were more sensitive to detecting wheeze in children than the clinician when matching both to automated spectrogram analysis [20]. The high sensitivity and improving accuracy of such devices underlines their potential benefit as valuable tools for (remote) diagnosis and monitoring in research settings [9]. However, there is a lack of published randomised controlled trials assessing the clinical efficacy of digital detectors of pathological airway sounds in the hands of patients and/or caretakers. Reviews examining the impact of digital interventions in children with asthma on clinical parameters have also shown inconsistent findings. A systematic review of digital asthma interventions found that half of the studies favoured digital interventions [9], while the other half reported no significant difference in asthma control.

Our results suggest that the wheeze detector used has no statistically significant clinical impact among this particular study population. However, trends in the results suggest that the impact of a digital device may be related to a variety of factors such as age, cultural background, disease severity, access to specialised healthcare providers or usage patterns.

Therefore, the chosen setting and study population might play an important role in studies evaluating clinical efficacy of developed technologies. For example, in a pilot study on the use of WheezeScan™, parents of 20 preschool children were instructed to use the device once every morning and evening in addition to when they felt it was needed. This led to a more frequent use than in the randomised controlled trial. Interestingly, the pilot study showed a positive trend in parental self-efficacy (PAMSES), although the absence of a control group and small sample size in the pilot study increases the risk of confounding factors. This observation underlines that in addition to standardised study protocols, it is essential to consider the characteristics of the target population when interpreting results.

### One size/device does not fit all

Potential factors influencing device efficacy have also been identified in the satisfaction and usability evaluation of the present study. The device usability was rated as uncomplicated by most parents, and most of them perceived their child to have benefitted from its use. This indicates a high degree of willingness to use an at-home digital support system among parents of children suffering from recurrent wheeze and is in line with current research in the field. However, several families reported difficulties, such as result variability, and found the device to be too sensitive to background noises. This perception may have impacted the willingness to use the wheeze detector frequently, which in turn may affect the power of outcome analyses. As real-life circumstances may have a significant impact on the use and effectiveness of digital devices, continued efforts to understand usage scenarios are key when evaluating the clinical effectiveness of digital support tools.

In addition, results on the perceived benefit varied not only between age groups, but also according to geographical and cultural background. A more critical evaluation and less frequent use of the device by patients from Berlin compared to Istanbul or London may imply that in addition to potential differences in the healthcare setting, cultural variations could affect aspects, such as perceived benefit and usage behaviour. Therefore, a deep understanding of the perception and usage of a specific digital tool among targeted patient groups is crucial in assessing digital health solutions.

### Strengths and limitations

The study's strengths include its randomised controlled trial design, enabling a more selective evaluation of the effects of the digital device on the various patient outcomes. A multicentre approach in different geographical and cultural settings, as well as the relatively large sample size compared to previous studies increase the generalisability of results.

However, the study also has important limitations as it focused on mildly to moderately affected children and excluded those with a more severe phenotype. Furthermore, the participants of the study were recruited in slightly different settings according to the study site. Whereas in Berlin and Istanbul children were recruited mainly from paediatric outpatient clinics, the study centre in London was a specialised clinic where participants were likely to have their first contact with a specialised paediatric pulmonologist. This may have affected the improvement of wheeze control and quality of life for all participants, independently from the study group. Finally, the recruitment period stretched over several seasons. Although the summer season was avoided by a recruitment break in July and August, the prevalence of wheezing exacerbations was relatively low and may have varied according to the time point of enrolment.

### Conclusions and perspectives for future research

The clinical evaluation of digital support tools to be used by patients and/or caretakers is crucial, as their clinical use may differ fundamentally from highly standardised research settings. The present study shows that despite its very good performance in previous validation studies, no significant clinical impact could be observed for the wheeze detector when tested in a multicentre randomised controlled trial. However, the study underlines the importance of further studies and a deeper understanding of parameters characterising the target group for digital support tools. Further research is needed to gain better insight into patient perception, usage behaviour and barriers to successful implementation.

## Supplementary material

10.1183/23120541.00518-2023.Supp1 **Please note:** supplementary material is not edited by the Editorial Office, and is uploaded as it has been supplied by the author.
Supplementary material 00518-2023.supplement

## References

[1] Doss AMA, Stokes JR. Viral infections and wheezing in preschool children. Immunol Allergy Clin North Am 2022; 42: 727–741. doi:10.1016/j.iac.2022.05.004 36265972

[2] Rajapakse Mudiyanselage SIR, Amarasiri W, Yasaratne B, et al. Epidemiology of wheeze among preschool children: a population-based cross-sectional study from rural Sri Lanka. BMJ Open 2021; 11: e046688. doi:10.1136/bmjopen-2020-046688 PMC8264903 34233982

[3] Herzog R, Cunningham-Rundles S. Pediatric asthma: natural history, assessment, and treatment. Mt Sinai J Med 2011; 78: 645–660. doi:10.1002/msj.20285 21913196 PMC3172616

[4] Tenero L, Tezza G, Cattazzo E, et al. Wheezing in preschool children. Early Hum Dev 2013; 89: Suppl. 3, S13–S17. doi:10.1016/j.earlhumdev.2013.07.017 PMC7130726 24001476

[5] Brand PL, Baraldi E, Bisgaard H, et al. Definition, assessment and treatment of wheezing disorders in preschool children: an evidence-based approach. Eur Respir J 2008; 32: 1096–1110. doi:10.1183/09031936.00002108 18827155

[6] Brand PL, Caudri D, Eber E, et al. Classification and pharmacological treatment of preschool wheezing: changes since 2008. Eur Respir J 2014; 43: 1172–1177. doi:10.1183/09031936.00199913 24525447

[7] Vasilopoulou I. Underdiagnosis and undertreatment of asthma in children: a tertiary hospital's experience. Clin Transl Allergy 2015; 5: P19. doi:10.1186/2045-7022-5-S2-P19

[8] Lozano P, Finkelstein JA, Hecht J, et al. Asthma medication use and disease burden in children in a primary care population. Arch Pediatr Adolesc Med 2003; 157: 81–88. doi:10.1001/archpedi.157.1.81 12517200

[9] Morrison D. Digital asthma self-management interventions: a systematic review. J Med Internet Res 2014; 16: e51. doi:10.2196/jmir.2814 24550161 PMC3958674

[10] Ferrante G, Licari A, Marseglia GL, et al. Digital health interventions in children with asthma. Clin Exp Allergy 2021; 51: 212–220. doi:10.1111/cea.13793 33238032 PMC7753570

[11] Alvarez-Perea A, Dimov V, Popescu FD, et al. The applications of eHealth technologies in the management of asthma and allergic diseases. Clin Transl Allergy 2021; 11: e12061. doi:10.1002/clt2.12061 34504682 PMC8420996

[12] Ramsey RR, Caromody JK, Voorhees SE, et al. A systematic evaluation of asthma management apps examining behavior change techniques. J Allergy Clin Immunol Pract 2019; 7: 2583–2591. doi:10.1016/j.jaip.2019.03.041 30954644 PMC6776707

[13] International Telecommunication Union. Report of the International Telecommunication Union (ITU). 2022. https://www.itu.int/en/ITU-D/Statistics/Pages/stat/default.aspx Date last accessed: 6 December 2023.

[14] Vasbinder EC, Goossens LM, Rutten-van Mölken MP, et al.. e-Monitoring of Asthma Therapy to Improve Compliance in children (e-MATIC): a randomised controlled trial. Eur Respir J 2016; 48: 758–767. doi:10.1183/13993003.01698-2015 27230437

[15] Merchant RK, Inamdar R, Quade RC. Effectiveness of population health management using the propeller health asthma platform: a randomized clinical trial. J Allergy Clin Immunol Pract 2016; 4: 455–463. doi:10.1016/j.jaip.2015.11.022 26778246

[16] Clark M, Romano C, Olayinka-Amao O, et al. Development and content validation of a self-completed, electronic Pediatric Asthma Symptom Diary. J Patient Rep Outcomes 2022; 6: 25. doi:10.1186/s41687-022-00432-3 35306621 PMC8934788

[17] Mayoral K, Garin O, Caballero MA, et al. Smartphone app for monitoring asthma in children and adolescents. Qual Life Res 2021; 30: 3127–3144 doi:10.1007/s11136-020-02706-z 33387290

[18] Hosseini A, Buonocore CM, Hashemzadeh S, et al. Feasibility of a secure wireless sensing smartwatch application for the self-management of pediatric asthma. Sensors (Basel) 2017; 17: 1780. doi:10.3390/s17081780 28771168 PMC5580199

[19] Sarasmita MA, Larasanty LPF, Kuo LN, et al. A computer-based interactive narrative and a serious game for children with asthma: development and content validity analysis. J Med Internet Res 2021; 23: e28796. doi:10.2196/28796 34515641 PMC8477291

[20] Kevat AC, Kalirajah A, Roseby R. Digital stethoscopes compared to standard auscultation for detecting abnormal paediatric breath sounds. Eur J Pediatr 2017; 176: 989–992. doi:10.1007/s00431-017-2929-5 28508991

[21] Urban C, Kiefer A, Conradt R, et al. Validation of the LEOSound® monitor for standardized detection of wheezing and cough in children. Pediatr Pulmonol 2022; 57: 551–559. doi:10.1002/ppul.25768 34800333

[22] Von Mutius E. Presentation of new GINA guidelines for paediatrics. The Global Initiative on Asthma. Clin Exp Allergy 2000; 30: Suppl. 1, 6–10. doi:10.1046/j.1365-2222.2000.00089.x. PMID:10849467. 10849467

[23] Murphy KR, Zeiger RS, Kosinski M, et al. Test for respiratory and asthma control in kids (TRACK): a caregiver-completed questionnaire for preschool-aged children. J Allergy Clin Immunol 2009; 123: 833–9.e9. doi:10.1016/j.jaci.2009.01.058 19348922

[24] Bursch B, Schwankovsky L, Gilbert J, et al. Construction and validation of four childhood asthma self-management scales: parent barriers, child and parent self-efficacy, and parent belief in treatment efficacy. J Asthma 1999; 36: 115–128. doi:10.3109/02770909909065155 10077141

[25] Juniper EF, Guyatt GH, Feeny DH, et al. Measuring quality of life in the parents of children with asthma. Qual Life Res 1996; 5: 27–34. doi:10.1007/BF00435966 8901364

[26] Fekkes M, Theunissen NC, Brugman E. Development and psychometric evaluation of the TAPQOL: a health-related quality of life instrument for 1–5-year-old children. Qual Life Res 2000; 9: 961–972. doi:10.1023/A:1008981603178 11284215

[27] Habukawa C, Ohgami N, Matsumoto N, et al. A wheeze recognition algorithm for practical implementation in children. PLoS ONE 2020; 15: e0240048. doi:10.1371/journal.pone.0240048 33031408 PMC7544038

[28] Dramburg S, Dellbrügger E, van Aalderen W, et al. The impact of a digital wheeze detector on parental disease management of pre-school children suffering from wheezing: a pilot study. Pilot Feasibility Stud 2021; 7: 185. doi:10.1186/s40814-021-00917-w 34627391 PMC8501322

[29] Lee SH, Kim YS, Yeo MK, et al. Fully portable continuous real-time auscultation with a soft wearable stethoscope designed for automated disease diagnosis. Sci Adv 2022; 8: eabo5867. doi:10.1126/sciadv.abo5867 35613271 PMC9132462

[30] Birring SS, Fleming T, Matos S, et al. The Leicester Cough Monitor: preliminary validation of an automated cough detection system in chronic cough. Eur Respir J 2008; 31: 1013–1018. doi:10.1183/09031936.00057407 18184683

