# Supplementary material for: Clinical efficacy and satisfaction of a digital wheeze detector in a multicentre randomised controlled trial: the WheezeScan study
Source: ERJ Open Res. 2024 Jan 15;10(1):00518-2023. doi: 10.1183/23120541.00518-2023 (PMC10789262; doi:10.1183/23120541.00518-2023)
Supplement: Supplementary file 1 [file 00518-2023.supplement.pdf]

Clinical efficacy and satisfaction of a digital wheeze detector in a multicenter randomized controlled trial – the WheezeScan study

- Electronic Repository –

Yen Hoang Do [1], Wim van Aalderen [2], Ellen Dellbrügger [3], Claude Grenzbach[3], Jonathan Grigg [4], Ulrike Grittner[5], Eric Haarman [2], Camilo José Hernandez Toro[1,5], Bulent Karadag[6], Siri Roßberg [3], Tina-Maria Weichert [3], Abigail Whitehouse [4], Antonio Pizzulli[1†], Paolo Maria Matricardi [1], Stephanie Dramburg [1*]

Authors' affiliations:

[1] Department of Pediatric Respiratory Medicine, Immunology and Critical Care Medicine, Charité - Universitätsmedizin Berlin.

[2] Department of Pediatric Respiratory Medicine and Allergy, Emma Children's Hospital, Amsterdam UMC, University of Amsterdam, Amsterdam, The Netherlands.

[3] Pediatric Pulmonologist, Berlin, Germany.

[4] Centre for Genomics and Child Health, Blizard Institute, Queen Mary University of London, London, United Kingdom.

[5] Institute of Biometry and Clinical Epidemiology, Charité - Universitätsmedizin Berlin.

[6] Division of Pediatric Pulmonology, Marmara University, Istanbul, Turkey.

Word count: 1.630
Figures: 9

[*]Corresponding author:
Stephanie Dramburg
Department of Pediatric Respiratory Medicine, Immunology and Critical Care Medicine
Charité Universitätsmedizin Berlin
Augustenburger Platz 1, 13353 Berlin
Tel.: +49-(0)30-450 559 389
stephanie.dramburg@charite.de

Methods

Study population - The parents and/or guardians of all participating children gave written informed consent regarding the child's participation in the study. In comparison to the study protocol, the age eligibility criteria for recruitment were adjusted by 12 months to include the full range of children in the pre-school age group.

Questionnaires - The safety outcomes regarding the emergency visits or hospital stays, and the unscheduled physician visits were recorded in the e-diary of the study app.

Digital Wheeze Detector - Parents were instructed to remove nasal mucous before the measurement and to create a calm environment to avoid confounding environmental sounds. In case of interference, e.g. by ambient sounds, the detector gives a visual error signal, and parents were instructed to repeat the measurement.

Study app - The mobile application WheezeMonitor® (TPS Software Production S.r.l, Rome, Italy) was used for recording respiratory symptoms, medication intake, and unscheduled visits to the doctor's office or emergency department as well as the results of the WheezeScan™ device via a daily electronic questionnaire. Automated daily reminders to fill the questionnaire were activated on the caretaker´s phone. After three consecutive days of missed reporting, parents were contacted by the study team via e-mail or phone call to check whether technical or other support was necessary.

Monitoring period - After the recruitment visit (T0) all participating families filled a daily questionnaire in the WheezeMonitor® app over 120 observation days. Questions addressed the child´s respiratory symptoms, medication intake, absence from day-care, missed workdays, and visits to the doctor´s office or emergency department. Families of the intervention group were additionally encouraged to use the digital wheeze detector whenever they felt that their child could be experiencing respiratory distress. The results of the device were also recorded via the study app.

Randomization - Randomization was performed by the study statistician Ulrike Grittner. The random assignment of the study participants into the two treatment groups (allocation ratio 1:1) was performed by blocked randomization with varying block size (block sizes: 2, 4, and 6) stratified by study center.

Sample size calculation - Based on the results of the pilot study [28] a sample size of 164 patients was calculated. In the pilot study the mean Asthma Control Test (ACT) score at baseline was 18.3 points (SD: 3.9), and 21.3 (SD: 4.4) after intervention. This variance measures corresponds to a pooled standard

deviation of 4.2. We expected a higher variance of 6 in this study, which is comparing two groups (intervention and control). The sample size was estimated assuming that the intervention group would have an improvement of the ACT score 3 points higher than the expected improvement with conventional treatment. A mean difference between intervention and control groups in ACT score of 3 points and a common standard deviation of 6 points corresponds to an effect size (Cohen's d) of 0.5. Although the outcome in the pilot study was not the TRACK score but the ACT, we assume similar effect sizes for the TRACK Score. Based on that assumption it was estimated that the enrolment of 164 children would provide 80% power with a two-sided 5% significance level allowing for a 22% dropout rate.

Missing data - Missing values in the data were assumed to be missing at random or completely at random. Thus, all missing values in the full analysis set were imputed using multiple imputation by chained equations with 30 imputed datasets and predictive mean matching to estimate values in a realistic range.

Multiple imputation methods - Multiple imputation by chained equations was performed with the mice package **[29]**. Estimated adjusted mean differences for the primary and secondary analysis were performed with lme4 **[30]** and emmeans **[31]** packages. All figures were produced using the ggplot2 **[32]** and ggeffects **[33]** packages.

Statistical analysis of secondary outcomes and subgroup results - Secondary analyses consisted of a comparison of the specific scores at the follow-up between intervention and control group using ANCOVA with the score as dependent variable and treatment group, center, and the respective score at T0 as covariates. Secondary analyses were done in an exploratory manner with no adjustment for multiple testing. For all secondary analyses, effect estimates, and corresponding 95%CI were estimated. Additionally, sensitivity analysis of the primary and secondary outcomes and safety analysis were conducted using the per-protocol data set (all participants, who recorded the study diary for at least 30% of the observation days between the study visits and attended T0, T1 and T2 visits within the scheduled timeframe +/- 3 days) **[Figure S1].**

Pre-specified subgroup analyses were performed using the full analysis set to identify probable differential treatment effects. Subgroups were defined by age at baseline (children of 4 to 12 months, 13 to 48 months, above 48 months of age at baseline), by severity of disease at baseline (children with controlled, TRACK at T0 >= 80 points, or uncontrolled, TRACK at T0 < 80 points, symptoms, and by study country were performed by testing an interaction term of subgroup by intervention in the specific regression models and marginal treatment effects and 95%CI per subgroup were estimated.

Safety rules

If a patient in the intervention group visited the emergency department or hospital two or more times during the study period, the intervention for that patient would have been stopped and the therapy regimen reevaluated. Additionally, if there was a misbalance of safety events that could be related to the intervention, the study would have been halted immediately for further investigation.

Results

Adverse events

No severe adverse events were reported, and all but one device functioned well. The malfunctioning device was replaced by a new one at the study center in Berlin.

Baseline differences between intervention and control group

While 31% (27/87) of the children in the intervention group suffered from allergy, this was only the case for 17.5% (14/80) of the controls. This difference was also reflected in a higher prevalence of positive skin prick test and/or serum IgE results and a more frequently indicated allergy among parents of the intervention group.

Subgroup analyses of the impact on wheezing control (TRACK)

When looking at TRACK scores at T1 by study country **[Figures e3 B, e4]**, differences become apparent with a larger intervention effect between treatment groups in Berlin and less or no differences in Istanbul or London. The subgroup analysis by asthma control (well controlled: TRACK at T0 ≥ 80 points; not well controlled: TRACK at T0 <80 points) showed a slight difference between treatment groups in the "not well controlled" subgroup, but no difference was seen in the "well controlled" subgroup **[Figure S2].**

A subgroup analysis by age groups (>48 months, 13-48 months, 4-12 months) revealed similar slight differences between treatment groups for children aged 13 months or older, while for younger infants, no differences between treatment groups could be observed (TRACK mean difference a) Age between 4 an 12 months: -5.71 (-26.05-14.63), Age between 13 and 48 months: 5.00 (-2.59-12.59), Age above 48 months (2.62, -8.44-13.67) **[Figure S3 C].**

Adherence to recording

During the complete monitoring period of 120 days, the median proportion of recorded days was 77% (Q1-Q3: 58%-94%) in the intervention and 80% (Q1-Q3: 68%-93%) in the control group. For more detailed information on the individual recording behavior, please see **[Figure S5]**.

The use of short-acting beta 2 agonists (SABA) was asked in the daily questionnaire and the participants required no SABA use during most days.

Device use

The frequency of device use was very heterogeneous, ranging between 1 and 255 times as recorded by the patients in the WheezeMonitor® app (median number of device usage 41, IQR 73). Differences in the usage behavior could be observed between study sites. For example, participants in Istanbul used the WheezeScan™ more frequently (individual patients up to 200 times, in the median participants used the device 78 times, IQR 94) while most patients from Berlin used it less than 50 times (median 28, IQR 74) **[Figure S6]**. The measurement results reflected a broad clinical heterogeneity ranging from 1-100% of positive ("wheeze") results per patient (median number of „wheeze" results 11, IQR 27.5) **[Figure S9B]**. However, high proportions of positive results were mainly observed among patients with low numbers of device use. No specific pattern could be observed for the occurrence of error signals regarding frequency of usage **[Figure S9A]**.

Device usability evaluation by study center

Notably, the proportion of participants without complications while using the device varied among the different trial sites. Specifically, in Berlin, only 66% of participants had no complications, while in Istanbul, 97% used the device without complications, and none of the participants in London had any complications with device usage. This also reflects in the satisfaction of the device at the different study centres. Only 24% (10/41) of the participants in Berlin think their child has benefitted from the device, much less than the participants in Istanbul (69%). In line with the information on challenges in device usage, the age group with the largest proportion of parents who perceived a benefit from using the WheezeScan™ was that with the oldest (>48 months) children **[Figure S7]**. Notably, more than 60% (45/75) of parents would continue to use the wheeze detecting device in the future and 64% (48/75) would recommend it to other parents whose children experience wheezing. When asked in an open question about their evaluation of the device, the high noise sensitivity of the device was mentioned repeatedly.

Discussion

Clinical efficacy of other types of digtal health interventions - Studies testing other types of digital health interventions in self-management or at home care settings have shown conflicting results regarding usability and clinical efficacy. For example, a study on a smart inhaler with remote monitoring and feedback features found no significant increase in asthma control **[37]**, while another study using a similar device with adherence reminders via SMS demonstrated improvement **[14]**.

Legends to the Figures

Figure S1 – Functional description of the digital wheeze detector

Figure S2 – Interventions effects for primary and main secondary endpoints for the per-protocol dataset at T1 (sensitivity analysis). A) **TRACK** mean difference (intervention vs. control ANCOVA): (2.6, 95%CI:-4.08-9.29, p=0.442); B) **PAMSES** mean difference (intervention vs. control ANCOVA): (-2.17, 95%CI:-4.95-0.61, p=0.125); C) **PACQLQ** mean difference (intervention vs. control ANCOVA): (0.18, 95%CI:-0.15-0.52, p=0.277); D) **TAPQLQ** mean difference (intervention vs. control ANCOVA): (-3.17, 95%CI:-6.66-0.31, p=0.074).

Figure S3 – TRACK mean difference (intervention vs. control, based on multiple imputation estimates and ANCOVA) by study group at T2 (A) and by B) study centres (Berlin (BER) $n^{control}$= 40 , $n^{intervention}$=42; Istanbul (IST) $n^{control}$=24 , $n^{intervention}$=31; London (LON) $n^{control}$=8 , $n^{intervention}$=5); C) age group (>48 months: $n^{control}$= 18 , $n^{intervention}$=15; 13-48 months $n^{control}$=44 , $n^{intervention}$=45; 4-12 months $n^{control}$=10 , $n^{intervention}$=18); and D) wheeze severity at T0 (mild = TRACK 80+; $n^{control}$= 24, $n^{intervention}$=19; severe = TRACK <80: $n^{control}$= 48 , $n^{intervention}$=59)

Figure S4 – Wheeze control (TRACK) at baseline (T0: baseline) and follow-up (T1: 90 days) by study centre (Berlin n=82, Istanbul n=55, London n=13)

Figure S5 – Individual adherence to e-diary reporting in the WheezeMonitor® app by study centre (Patients with App records and without problematic e-diary entries: Berlin n=81, Istanbul n=57, London n=20). Red line: end of first monitoring period (90 days), green line: end of complete monitoring period (120 days)

Figure S6 – Distribution of device use by study centre (Berlin n=85, Istanbul n=59, London n=23) as recorded via the study app

Figure S7 – Perceived benefit of device use as reported by parents after 90 days of usage

Figure S8 – Daily use of short-acting beta 2 agonists (SABA) as reported in the study app by study group (control group n=77, intervention group n=81).

Figure S9 – A) Total number and results of measurements with the WheezeScan™ device as reported in the study app (intervention group with valid WheezeScan™ measurements recorded in WheezeMonitor® n=81) during the whole monitoring period (until T2). **B)** Proportion of measurement results per participant of the intervention group (intervention group with valid WheezeScan™ measurements recorded in WheezeMonitor® n=81) during the whole monitoring period (until T2).

Figure S1

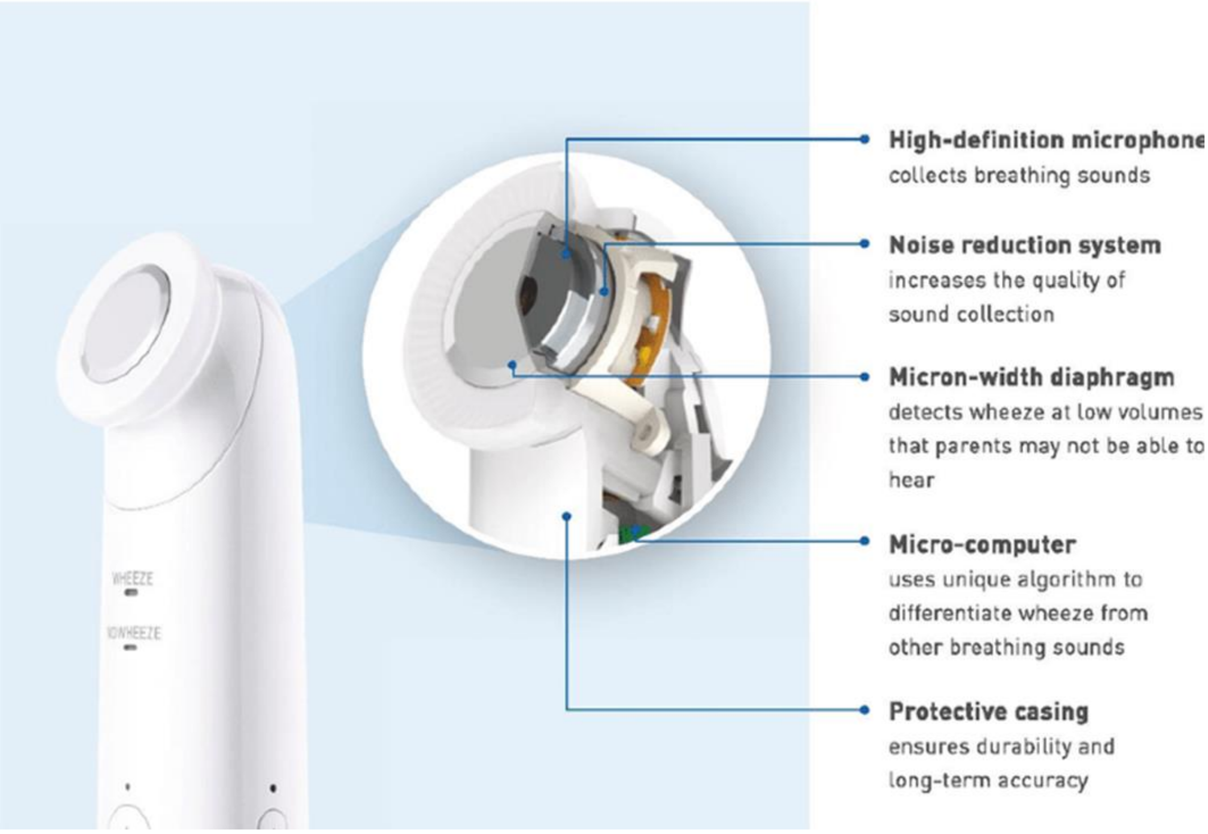
WHEEZE
NOWHEEZE
High-definition microphone
collects breathing sounds
Noise reduction system
increases the quality of
sound collection
Micron-width diaphragm
detects wheeze at low volumes
that parents may not be able to
hear
Micro-computer
uses unique algorithm to
differentiate wheeze from
other breathing sounds
Protective casing
ensures durability and
long-term accuracy

Figure S2

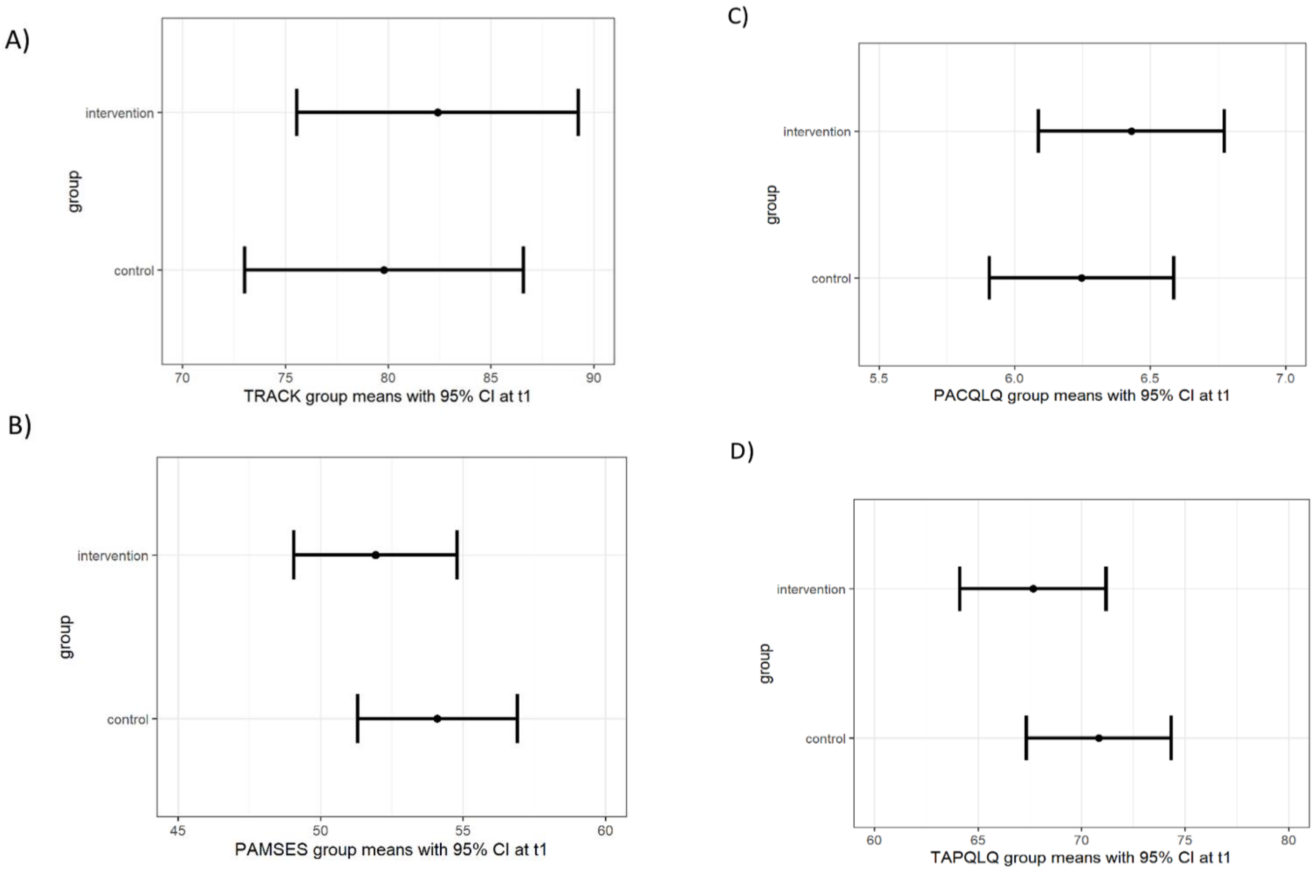
A)
intervention
control
group
70
75
80
85
90
TRACK group means with 95% CI at t1
B)
intervention
control
group
45
50
55
60
PAMSES group means with 95% CI at t1
C)
intervention
control
group
5.5
6.0
6.5
7.0
PACQLQ group means with 95% CI at t1
D)
intervention
control
group
60
65
70
75
80
TAPQLQ group means with 95% CI at t1

Figure S3

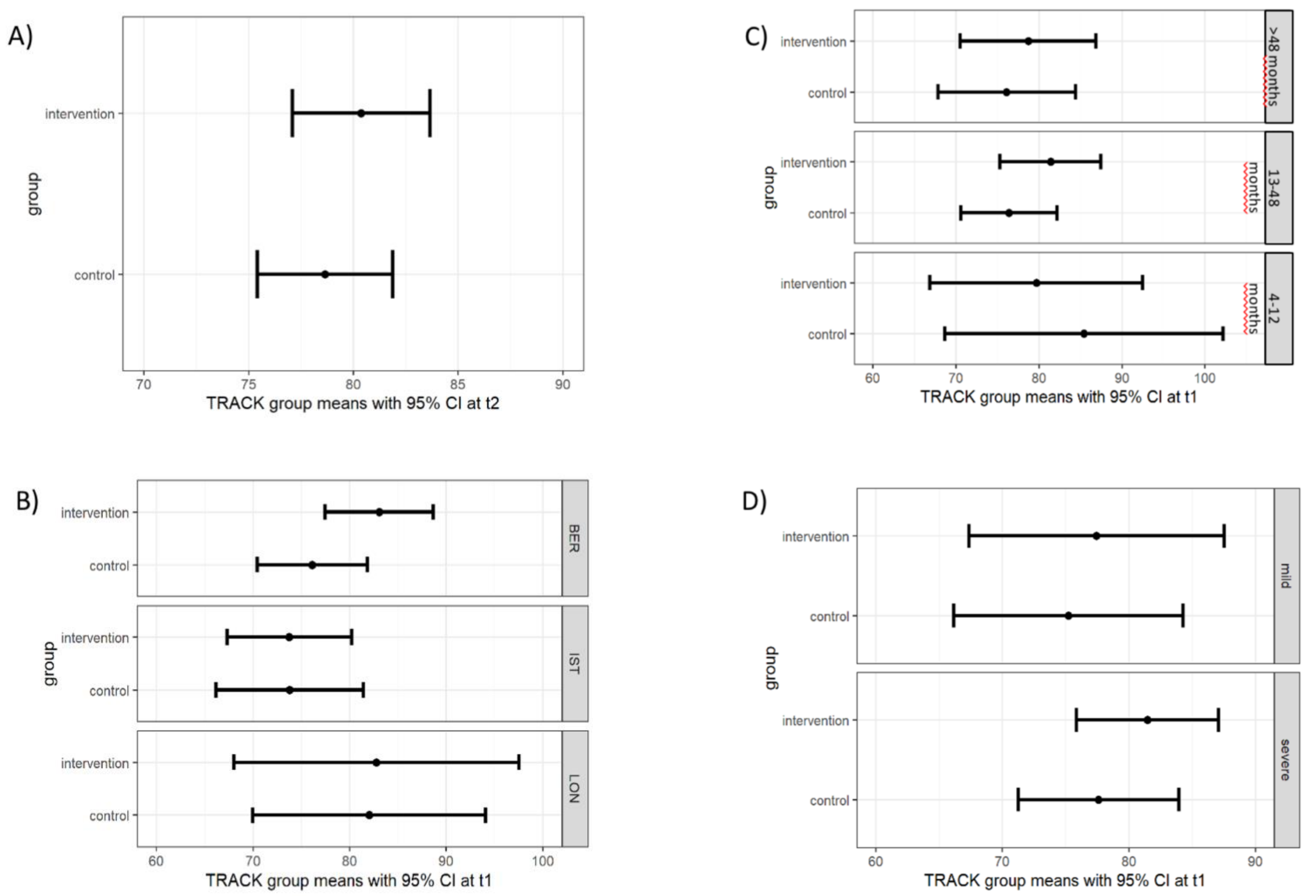
A)
intervention
control
group
70
75
80
85
90
TRACK group means with 95% CI at t2
B)
BER
IST
LON
intervention
control
group
60
70
80
90
100
TRACK group means with 95% CI at t1
C)
>48 months
13-48 months
4-12 months
intervention
control
group
60
70
80
90
100
TRACK group means with 95% CI at t1
D)
mild
severe
intervention
control
group
60
70
80
90
TRACK group means with 95% CI at t1

Figure S4

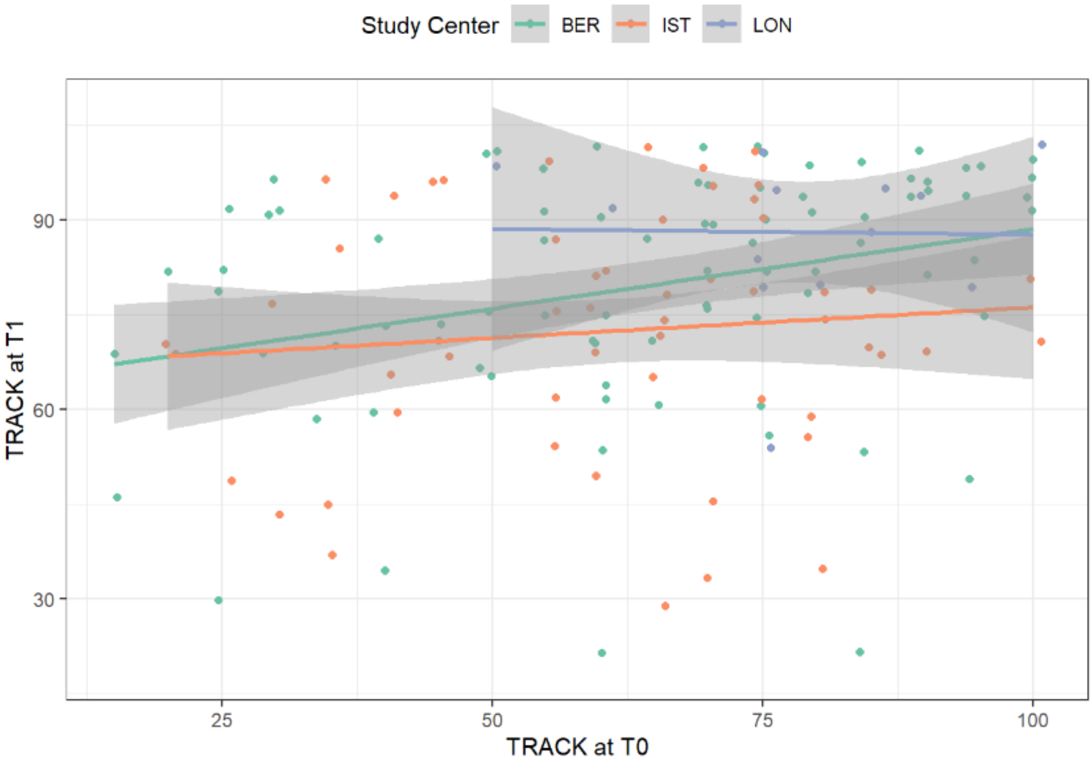

Study Center
BER
IST
LON
90
60
30
TRACK at T1
25
50
75
100
TRACK at T0

Figure S5

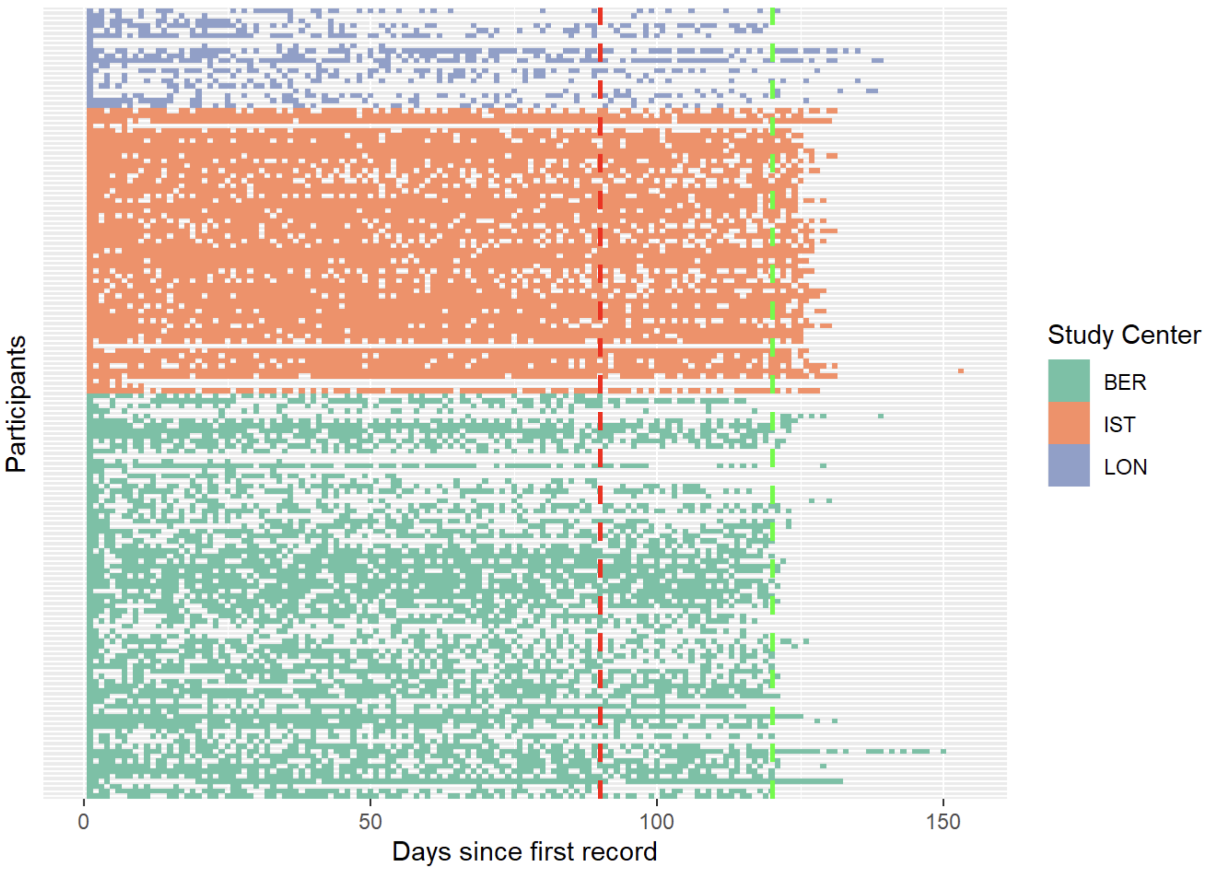
Participants
0
50
100
150
Days since first record
Study Center
BER
IST
LON

Figure S6

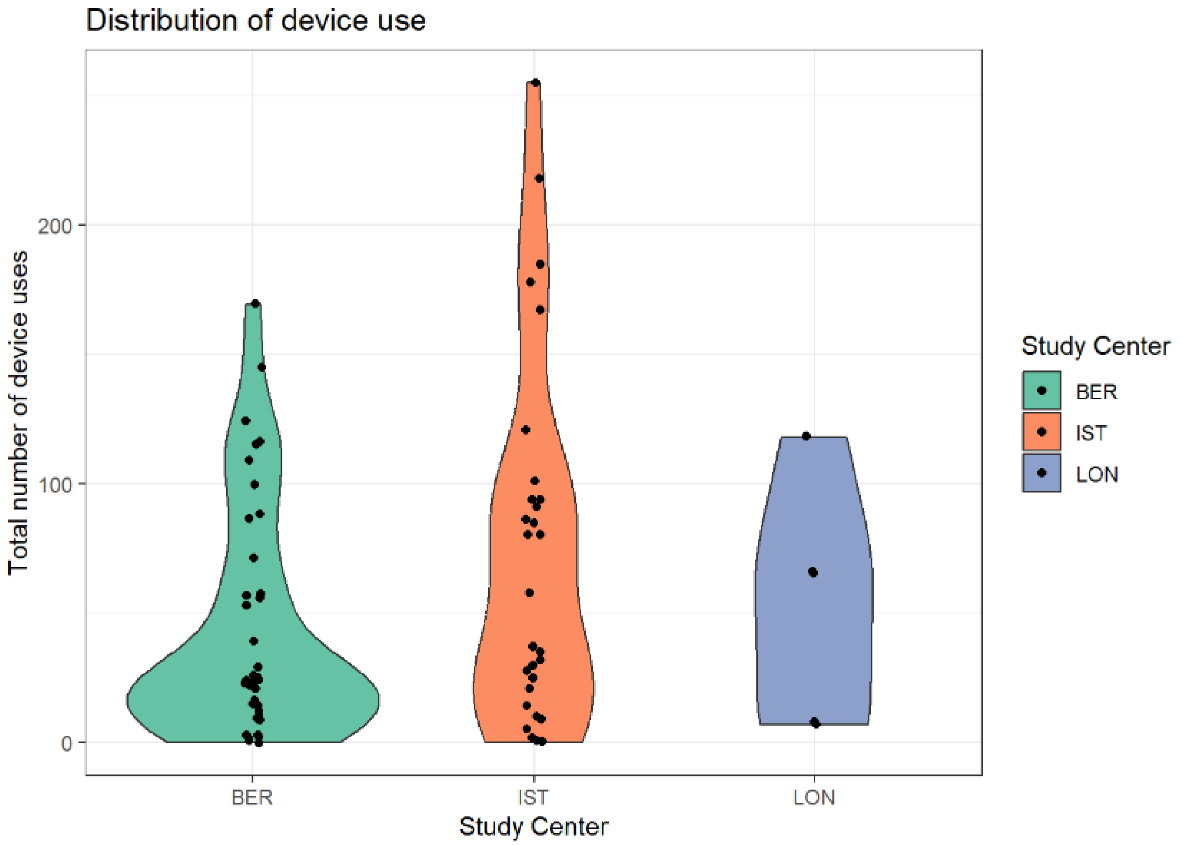

Distribution of device use
Total number of device uses
0
100
200
BER
IST
LON
Study Center
Study Center
BER
IST
LON

Figure S7

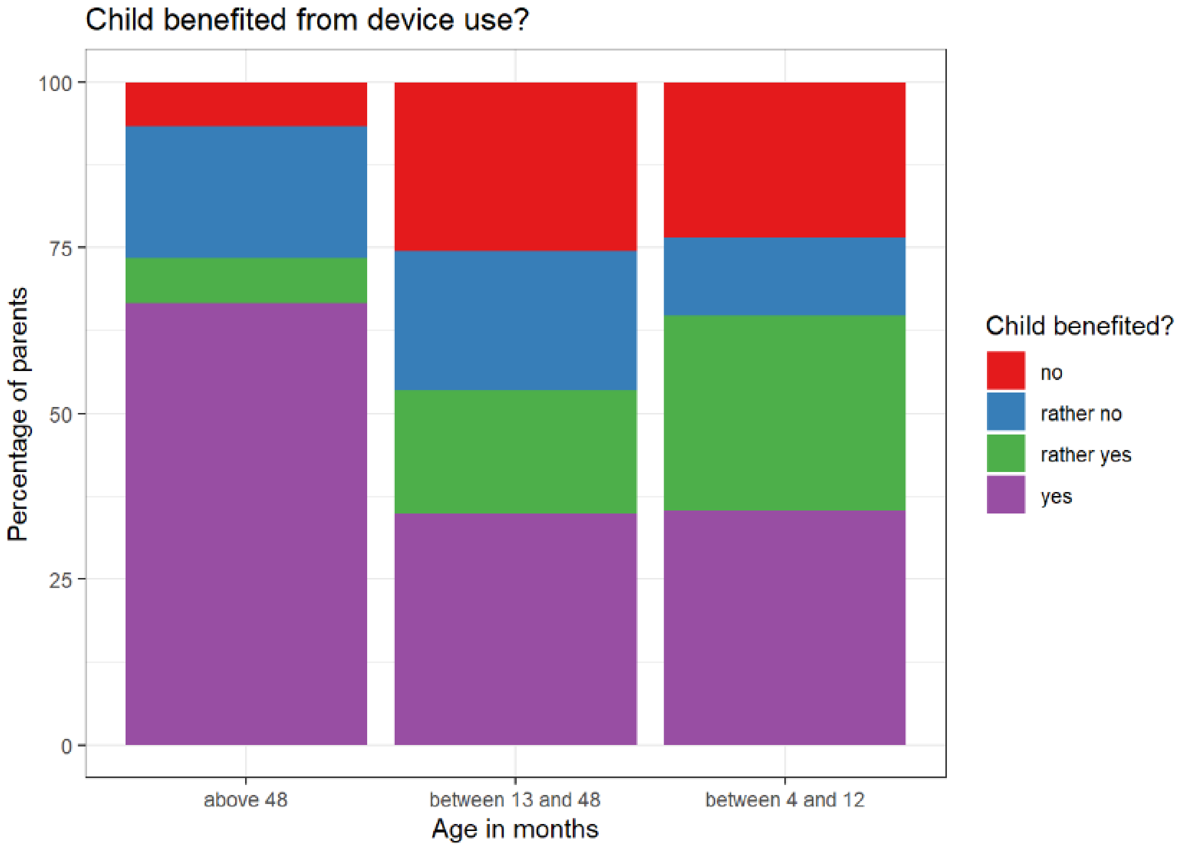
Child benefited from device use?
100
75
50
25
0
Percentage of parents
above 48
between 13 and 48
between 4 and 12
Age in months
Child benefited?
no
rather no
rather yes
yes

Figure S8

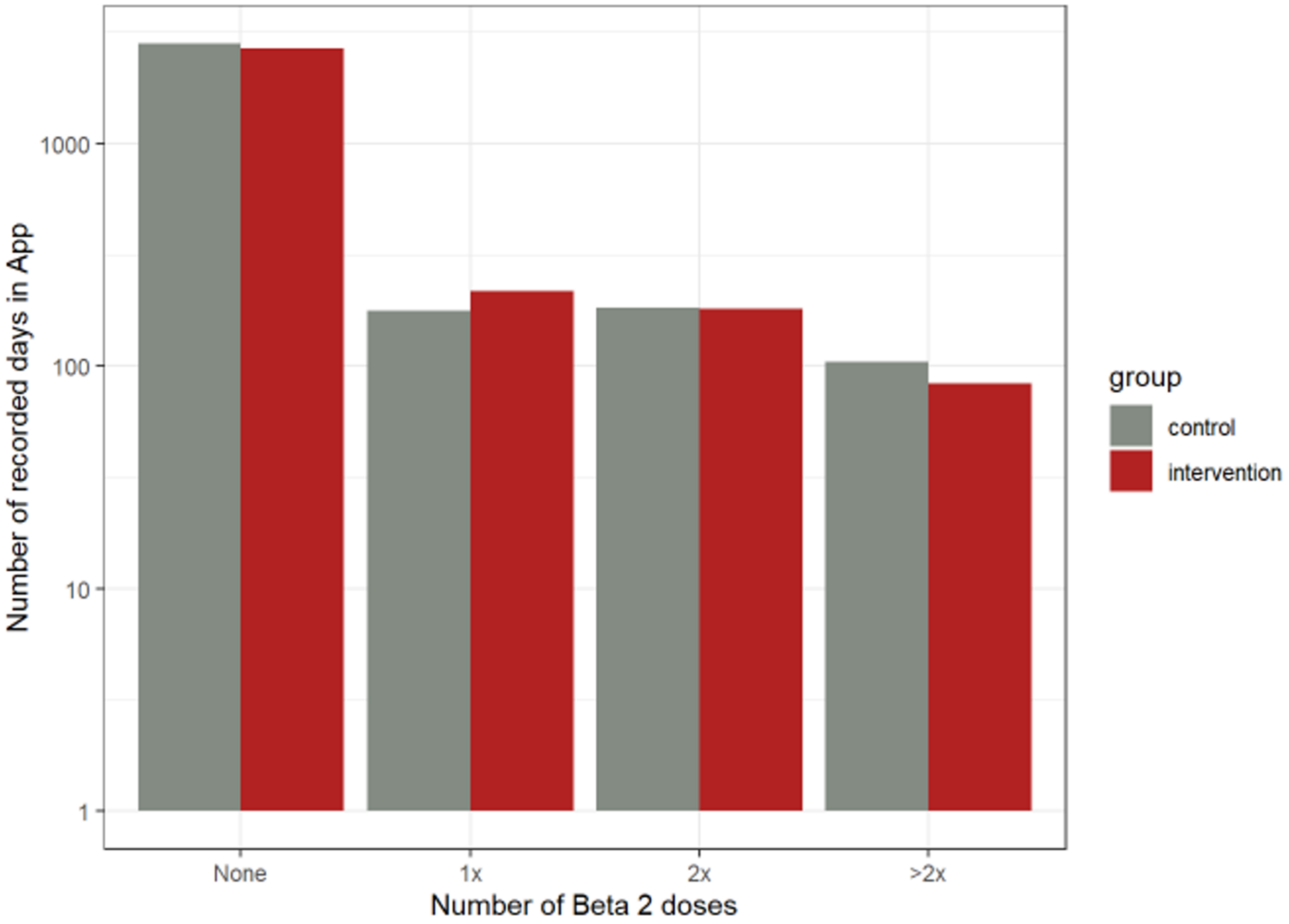
1000
100
10
1
Number of recorded days in App
None
1x
2x
>2x
Number of Beta 2 doses
group
control
intervention

Figure S9

A)

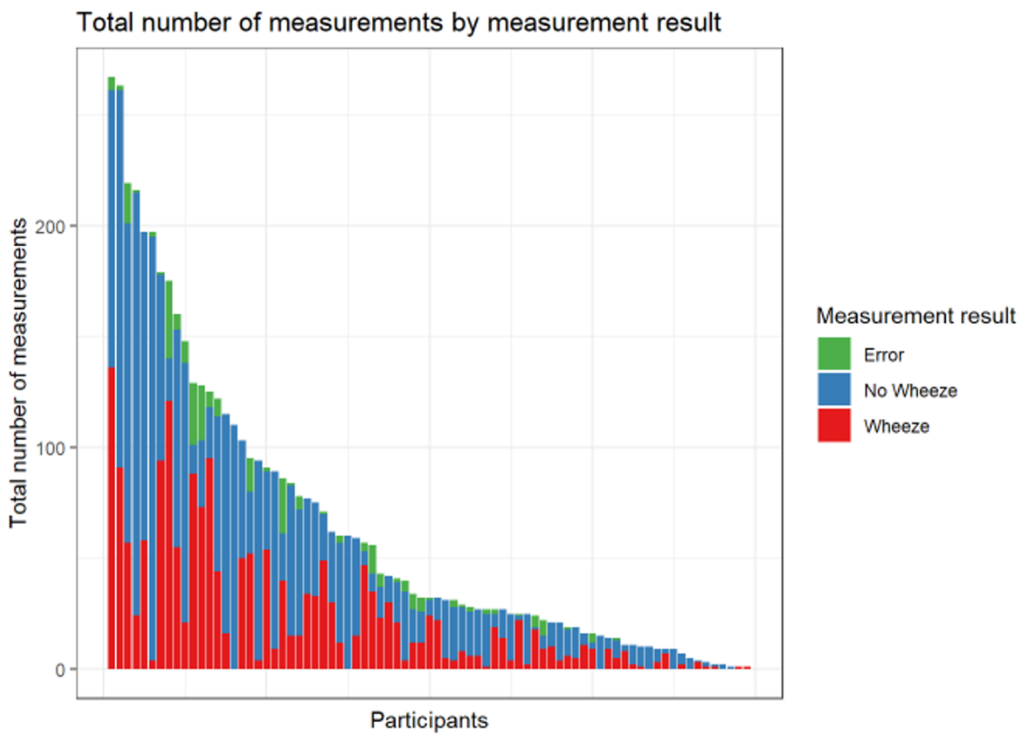

Total number of measurements by measurement result
Total number of measurements
200
100
0
Participants
Measurement result
Error
No Wheeze
Wheeze

B)

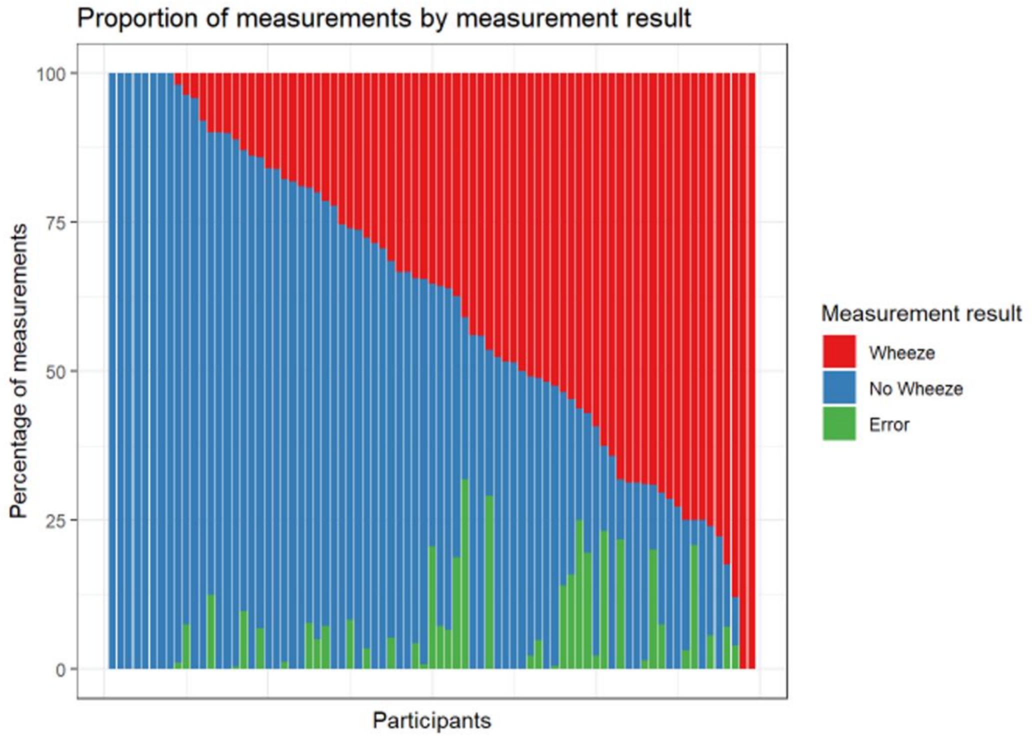

Proportion of measurements by measurement result
Percentage of measurements
100
75
50
25
0
Participants
Measurement result
Wheeze
No Wheeze
Error
